# Columbus City Courier

**Published:** 1856-04

**Authors:** 


### BIBLIOGRAPHICAL NOTICES.

*Columbus City Courier.*—This is the title of a secular paper, projected in Louisa County in this State, and edited by our friend B. G. Neal, M. D.

The Doctor will doubtless make this a valuable family paper, as his talents and acquirements eminently qualify him for the task. He will, doubtless, make it the medium through which much valuable information, on medical subjects, can be made to reach the masses. Medical journals do not reach these, and therefore they become the prey to quackery and the victims of empiricism. We would wish more of our secular journals were edited by scientific medical men, and humanity would be saved the pangs it is often made to suffer, through ignorance and blind credulity. We trust the Doctor will devote a page or two to the discussion of medical subjects, as they are always interesting to the reading public. We have been surprised at the apathy manifested byt he press on these subjects, as their papers would be better sustained, if they would extract occasionally from Medical Journals, for their columns. We hope our friend may be successful in his enterprise, and that he will exchange with us.

---

There now! Only in our last number did we give notice of this marriage conjunction, and, like many other matches, was made to be broken. What is the matter now? A blissful honeymoon has scarcely passed, until a separation takes place, and the groom retires, sets up for himself, and resumes his "benedict" name, which in these days of woman's rights, it seems, he surrendered. The "sober second thought" taught him to spurn the innovation, and in affection for time-honored usage, he proclaims himself "Richard again." They must not complain if this circumstance should give rise to some gossip in certain circles, for all such incidents are fruitful of speculation, and in a particular manner, if the marriage has been made between high contracting parties, or as our friend of the Gazette says, "in high life." We are advised that the "Stethoscope" must hereafter be known as the "Monthly Stethoscope and Medical Reporter," which, in future, we will be careful to observe, out of the highest respect and consideration for that periodical.

We have received the following works from *Blanchard & Lea*, publishers, at Philadelphia, but having received them too late for notice in this number, they will be considered in the next. We would take this occasion to say that we will have catalogues of recent works, which may not be found in our ample book stores, and our friends desiring works of recent medical literature, can be supplied through the editors of this Journal.

Parrish's Pharmacy; Sargent's Miner Surgery; Budd on Stomach; Barlow's Practice; Miller's Principles; Neill and Smith's Compendium of Medicine.

